# Supplementary material for: Factors Impacting COVID-19 Vaccine Uptake and Confidence Among Immigrant and Refugee Populations in Canada
Source: Int J Environ Res Public Health. 2025 Mar 26;22(4):493. doi: 10.3390/ijerph22040493 (PMC12027058; doi:10.3390/ijerph22040493)
Supplement: Supplementary file 1 [file ijerph-22-00493-s001.zip › ijerph-3524651-supplementary.pdf]

ASSOCIATION CANADIENNE DES CENTRES DE SANTÉ COMMUNAUTAIRE

CANADIAN ASSOCIATION OF COMMUNITY HEALTH CENTRES

CACHC Key Informant Interview: Vaccine Promotion Promising Practices

1. How did your CHC identify the need for this intervention(s)?

2. Who was involved in developing this intervention(s)?

3. What planning (framework/strategies) needed to be put in place before delivering the program?

4. Who delivered the intervention(s)?

5. What changes have you observed as a result of this intervention(s)?

6. Was any data collected to identify the effectiveness and outcome(s) of the program?

7. Can this program be replicated outside your CHC or scaled?

8. Are there any lessons learned from your experiences of using this (promising practice) for vaccine promotion and/or administration?

9. Has this intervention(s) been implemented among children 5 – 11 years and their caregivers?

10. Has your CHC developed or implemented any other promising vaccine educational or promotional programs for children 5 – 11 years and their caregivers?
